# Inpatient management of children with severe acute malnutrition: a review of WHO guidelines

**DOI:** 10.2471/BLT.15.162867

**Published:** 2016-07-08

**Authors:** Kirkby D Tickell, Donna M Denno

**Affiliations:** a Department of Epidemiology, University of Washington, Seattle, United States of America (USA).; b Departments of Pediatrics and Global Health, University of Washington, 6200 NE 74th Street, Suite 110, Box 354920, Seattle, WA 98115, USA.

## Abstract

**Objective:**

To understand how the World Health Organization’s (WHO’s) guidelines on the inpatient care of children with complicated severe acute malnutrition may be strengthened to improve outcomes.

**Methods:**

In December 2015, we searched Google scholar and WHO’s website for WHO recommendations on severe acute malnutrition management and evaluated the history and cited evidence behind these recommendations. We systematically searched WHO International Clinical Trials Registry Platform, clinicaltrials.gov and the Controlled Trials metaRegister until 10 August 2015 for recently completed, ongoing, or pending trials.

**Findings:**

WHO’s guidelines provide 33 recommendations on the topic. However, 16 (48.5%) of these recommendations were based solely on expert opinion – unsupported by published evidence. Another 11 (33.3%) of the recommendations were supported by the results of directly relevant research – i.e. either randomized trials (8) or observational studies (3). The other six recommendations (18.2%) were based on studies that were not conducted among children with complicated severe malnutrition or studies of treatment that were not identical to the recommended intervention. Trials registries included 20 studies related to the topic, including nine trials of alternative feeding regimens. Acute medical management and follow-up care studies were minimally represented.

**Conclusion:**

WHO’s guidelines on the topic have a weak evidence base and have undergone limited substantive adjustments over the past decades. More trials are needed to make that evidence base more robust. If the mortality associated with severe malnutrition is to be reduced, inpatient and post-discharge management trials, supported by studies on the causes of mortality, are needed.

## Résumé

**Objectif:**

Comprendre comment renforcer les lignes directrices de l'Organisation mondiale de la Santé (OMS) relatives à la prise en charge des enfants hospitalisés pour malnutrition aiguë sévère avec complications en vue d’améliorer les résultats.

**Méthodes:**

En décembre 2015, nous avons recherché les recommandations de l'OMS concernant la prise en charge de la malnutrition aiguë sévère dans Google Scholar et sur le site Internet de l'OMS, puis évalué l'historique et les éléments invoqués à l'appui de ces recommandations. Nous avons systématiquement recherché les essais récemment effectués, en cours ou en attente, jusqu'au 10 août 2015, dans le Système d'enregistrement international des essais cliniques de l'OMS, sur ClinicalTrials.gov et dans le metaRegister of Controlled Trials.

**Résultats:**

Les lignes directrices de l'OMS contiennent 33 recommandations à ce sujet. Cependant, 16 (48,5%) d'entre elles s'appuyaient uniquement sur une opinion d'expert non étayée par des données publiées. Onze (33,3%) autres étaient corroborées par les résultats de recherches présentant un intérêt direct, c'est-à-dire des essais randomisés (8) ou des études observationnelles (3). Les six dernières (18,2%) reposaient quant à elles sur des études n'ayant pas été menées auprès d'enfants atteints de malnutrition sévère avec complications ou sur des études de traitement non conforme à l'intervention recommandée. Les essais enregistrés incluaient 20 études en lien avec le sujet, dont neuf essais de régimes alimentaires alternatifs. Les études sur la prise en charge médicale urgente et les soins de suivi n'étaient que très peu représentées.

**Conclusion:**

Les lignes directrices de l'OMS sur le sujet s'appuient sur des données insuffisantes et n'ont fait l'objet que d'ajustements substantiels limités au cours des dernières décennies. Il est nécessaire de réaliser davantage d’essais afin de rendre cet ensemble de données plus fiable. Pour réduire la mortalité associée à la malnutrition sévère, il est nécessaire de réaliser des essais sur la prise en charge des enfants pendant et après leur hospitalisation, et de les étayer par des études sur les causes de la mortalité.

## Resumen

**Objetivo:**

Comprender cómo deben fortalecerse las directrices de la Organización Mundial de la Salud (OMS) sobre la atención hospitalaria de niños con malnutrición aguda grave complicada con el fin de mejorar los resultados.

**Métodos:**

En diciembre de 2015, se realizaron búsquedas de recomendaciones eruditas en Google y en el sitio web de la OMS en relación con la gestión de la malnutrición aguda grave y se evaluó el historial y se citaron las pruebas detrás de estas recomendaciones. De forma sistemática, se realizaron búsquedas de ensayos completados, en proceso o pendientes hasta el 10 de agosto de 2015 en la Plataforma Internacional de Registros de Ensayos Clínicos (International Clinical Trials Registry Platform) de la OMS, en clinicaltrials.gov y en el Metarregistro de Ensayos Clínicos Controlados (Controlled Trials metaRegister).

**Resultados:**

Las directrices de la OMS ofrecen 33 recomendaciones sobre el tema. No obstante, 16 (48,5%) de estas recomendaciones se basaron únicamente en opiniones de expertos, sin el respaldo de pruebas publicadas. Otras 11 (33,3%) recomendaciones estaban respaldadas por los resultados de investigaciones directamente relevantes, es decir, ensayos aleatorizados (8) o estudios de observación (3). Las otras 6 recomendaciones (18,2%) se basaban en estudios que no se realizaron en niños con malnutrición grave complicada o en estudios de tratamientos que no eran idénticos a la intervención recomendada. Los registros de los ensayos incluían 20 estudios relacionados con el tema, incluyendo 9 ensayos de regímenes de alimentación alternativos. La gestión médica aguda y los estudios de casos de seguimiento obtuvieron una representación mínima.

**Conclusión:**

Las directrices de la OMS sobre el tema tienen una base de pruebas deficiente y han sufrido pocos ajustes importantes durante las últimas décadas. Es preciso realizar más ensayos para que esta base de pruebas sea mucho más firme. Si se pretende reducir la mortalidad asociada a la malnutrición grave, se necesitan ensayos de gestión hospitalaria y tras el alta, con el apoyo de estudios basados en las causas de la mortalidad.

## ملخص

**الغرض:**

فهم كيف يمكن تعزيز المبادئ التوجيهية الخاصة بمنظمة الصحة العالمية حول رعاية المرضى من الأطفال المصابين بحالات معقدة من سوء التغذية الحاد والشديد داخل المستشفى من أجل تحسين المحصّلات.

**الطريقة:**

في شهر ديسمبر من عام 2015، قمنا بالبحث في الباحث العلمي من Google scholar والموقع الإلكتروني الخاص بمنظمة الصحة العالمية لمعرفة توصيات منظمة الصحة العالمية بشأن إدارة حالات الإصابة بسوء التغذية الحاد والشديد وقمنا بتقييم التاريخ والأدلة الواردة نتيجة لهذه التوصيات. وبحثنا بشكل منهجي في منبر منظمة الصحة العالمية للسجلات الدولية للتجارب السريرية، وهو clinicaltrials.gov والسجل العام "metaRegister" للتجارب المضبطة بالشواهد حتى 10 أغسطس 2015 للتجارب المكتملة مؤخرًا أو الجارية أو المعلّقة.

**النتائج:**

توفر المبادئ التوجيهية الخاصة بمنظمة الصحة العالمية 33 توصية بشأن الموضوع. ومع ذلك، استندت 16 توصية من هذه التوصيات (بنسبة ‏48.5%) على آراء الخبراء وحدها، غير مدعومة من جانب الأدلة المنشورة. وتم دعم 11 توصية أخرى من التوصيات بنسبة (33.3‏%) من جانب نتائج البحوث ذات الصلة المباشرة، أي إما التجارب المعشاة (8) أو الدراسات الرصدية (3). استندت التوصيات الست الأخرى (بنسبة 18.2‏%) على دراسات لم يتم إجراؤها بين الأطفال المصابين بحالات معقدة من سوء التغذية الشديد أو دراسات العلاج التي لم تكن مماثلة للتدخل المُوصى به. اشتملت سجلات التجارب على 20 دراسة متعلقة بالموضوع، من بينها تسع تجارب لأنظمة التغذية البديلة. كانت الدراسات الخاصة بإدارة الحالات الطبية الحادة والرعاية اللاحقة للمرضى ممثلة بالحد الأدنى.

**الاستنتاج:**

يوجد لدى المبادئ التوجيهية الخاصة بمنظمة الصحة العالمية قاعدة ضعيفة من الأدلة وخضعت لبعض التعديلات الموضوعية المحدودة على مدار العقود الماضية. وهناك حاجة لإجراء المزيد من التجارب لتعزيز قاعدة الأدلة. إذا كان معدل الوفيات المرتبط بسوء التغذية الحاد في طريقه للانخفاض، فهناك حاجة إلى إجراء تجارب إدارة المرضى داخل المستشفى وبعد الخروج من المستشفى، مدعومة بدراسات حول أسباب الوفيات.

## 摘要

**目的:**

了解如何加强世界卫生组织 (WHO) 严重营养不良儿童住院治疗指南，以改善治疗结果。

**方法:**

2015 年 12 月，我们在谷歌学术和 WHO 网站上搜索了关于严重急性营养不良管理的 WHO 建议，并且评估了这些建议背后的历史和引用的证据。 我们在 2015 年 8 月 10 日之前在 WHO 国际临床试验注册平台 (clinicaltrials.gov) 和临床对照试验 (mRCT) 上进行了系统性搜索，以查找最近完成的、正在进行的或即将进行的试验。

**结果:**

WHO 指南针对这一课题提出 33 条建议。 然而，这些建议中，有 16 条 (48.5%) 仅基于专家意见——无公开发表的证据支持。 另外 11 条建议 (33.3%) 基于直接相关研究的结果——即随机试验 (8) 或观察性研究 (3)。 还有 6 条建议 (18.2%) 基于未在复杂严重营养不良儿童身上进行的研究或不同于所建议干预措施的治疗研究。 试验注册平台上包含 20 项与本课题相关的研究，包括 9 项替代性供餐计划试验。 关于急性医疗管理和后续护理的研究数量很少。

**结论:**

WHO 指南在该课题证据基础薄弱，并且在过去数十年仅进行了有限的实质性调整。 需要进行更多试验以增强证据基础。 要想降低与严重营养不良相关的死亡率，我们需要进行由死亡原因研究支持的住院和出院后管理试验

## Резюме

**Цель:**

Определить возможные способы усовершенствования руководящих принципов Всемирной организации здравоохранения (ВОЗ) по ведению детей, страдающих тяжелой острой недостаточностью питания с осложнениями, в условиях стационара для улучшения конечных результатов.

**Методы:**

В декабре 2015 года авторы статьи провели поиск в системе Google Scholar и на веб-сайте ВОЗ на предмет рекомендаций ВОЗ по ведению тяжелой острой недостаточности и проанализировали историю изменений этих рекомендаций и цитируемые факты, лежащие в их основе. До 10 августа 2015 года также выполнялся систематический поиск на Международной платформе для регистрации клинических испытаний (International Clinical Trials Registry Platform) ВОЗ, clinicaltrials.gov, а также в Метарегистре контролируемых исследований (Controlled Trials metaRegister) на предмет недавно завершенных, текущих или еще не завершенных исследований.

**Результаты:**

Руководящие принципы ВОЗ содержат 33 рекомендации по данной теме. Однако 16 (48,5%) из этих рекомендаций основывались исключительно на экспертном мнении, не подтвержденном опубликованными фактическими данными. Еще 11 (33,3%) из этих рекомендаций были подкреплены результатами непосредственно относящегося к ним исследования, рандомизированного (8) или наблюдательного (3). Остальные шесть рекомендаций (18,2%) основывались на исследованиях, которые проводились не среди детей с осложненной тяжелой недостаточностью питания, или на исследованиях лечения, которое не было идентично рекомендуемому вмешательству. Реестры исследований включали 20 исследований, относящихся к данной теме, в том числе девять исследований альтернативных рационов. Исследования неотложной медицинской помощи и последующего ухода были практически не представлены.

**Вывод:**

Руководящие принципы ВОЗ по данной теме имеют слабо подкрепленную доказательную базу, и их содержание незначительно изменялось в течение последних десятилетий. Необходимо выполнить больше исследований для того, чтобы укрепить соответствующую доказательную базу. Для снижения смертности, связанной с тяжелой недостаточностью питания, потребуются исследования ведения пациентов в условиях стационара и наблюдения после выписки, подкрепленные изучением причин смертности.

## Introduction

Each year, severe acute malnutrition – defined as a weight-for-height *z*-score of less than  −3 or a mid-upper arm circumference of less than 115 mm – is the direct cause of an estimated 540 000 child deaths and an important underlying contributor to many other child deaths, especially those due to pneumonia and diarrhoea.^1^^,^^2^ The prevalence of – and case fatality rate for – malnutrition are particularly high in infants.^3^^,^^4^ Severe acute malnutrition without medical complications can now be effectively managed in the community, with ready-to-use therapeutic foods.^5^ The presence of complications such as anorexia, infections or metabolic dysfunction still warrants inpatient management. The World Health Organization (WHO) indicates that, by following its inpatient management guidelines, less than 10% of children with complicated severe acute malnutrition should die.^2^ However, despite reported compliance with these guidelines, health centres in sub-Saharan Africa have reported mortality rates of 10–40% among severely malnourished hospitalized children.^3^^,^^6^ The corresponding published rates in Asia tend to be lower, possibly because: Asian health systems are generally stronger than their African counterparts; therapeutic innovations have been introduced in some Asian facilities that have not yet been used in Africa; and children with uncomplicated severe malnutrition are sometimes admitted to Asian health facilities – but not, generally, to African health facilities.^7^^,^^8^ In sub-Saharan Africa, human immunodeficiency virus (HIV) is believed to contribute greatly to malnutrition-related mortality – although, a recent meta-analysis demonstrated an overall case fatality rate of 15% among paediatric inpatients with severe malnutrition without HIV infection.^6^

It is unlikely that severe acute malnutrition will be eliminated in the foreseeable future, as preventative interventions would have to reach the 52 million children who have moderate acute malnutrition.^1^ Optimizing the management of complicated severe malnutrition therefore remains an important strategy for reducing malnutrition-related mortality.

WHO’s first guidelines on the management of malnutrition – published in 1981 and focused on protein-energy malnutrition^9^– were replaced in 1999 by guidelines on the management of severe acute malnutrition.^10^ These two documents summarized decades of clinical experience and described the achievement of low malnutrition-related case fatality rates in some specific settings.^9^^-^^11^ Further guideline revisions were made in 2003^2^ and 2013.^4^ Relevant joint statements from WHO and other United Nations agencies were issued in 2007^5^ and 2009.^12^ The combination of these joint statements, the 1999 guidelines and the revisions of 2003 and 2013 constitutes the current WHO severe acute malnutrition guidelines and underpins WHO’s related training material.^13^

Although weak health systems and the inadequate implementation of guidelines undoubtedly contribute to the high number of preventable deaths attributed to complicated severe acute malnutrition,^14^ this condition causes high case fatality even in relatively well resourced centres that report full implementation of the WHO guidelines. This review attempts to identify evidence gaps within the guidelines on inpatient management of severe acute malnutrition that, if filled, may help reduce mortality below the levels that can be accomplished solely by adherence to existing guidelines. We reviewed each individual recommendation contained within current WHO guidelines – including those relating to the post-discharge care that forms an integral extension of hospital management. We traced the lineage and quantified the evidence cited in support of each recommendation. We also searched trials registries systematically, to determine which evidence gaps may be closed by the results of ongoing or recently completed trials.

## Methods

We identified WHO’s recommendations for severe acute malnutrition management by searching Google Scholar – using “severe acute malnutrition” and “author:WHO” as the search terms – and by downloading the publications on the WHO nutrition website in December 2015.^15^ Full texts were reviewed if they represented a relevant guideline – as classified by WHO’s guideline review committee – or guideline update.^16^ Documents specific to humanitarian crises and those without inpatient or post-discharge management recommendations – e.g. the 2007 and 2009 joint statements^5^^,^^12^ – were excluded. Each included guideline was parsed into individual recommendations. We excluded diagnosis and admission criteria and care principles that are applicable to all hospitalized children – e.g. the monitoring of blood glucose after treatment of hypoglycaemia.

We traced each recommendation’s evolution through the development of the guidelines and noted any modifications and references cited in support of the recommendation. The full texts of all potentially relevant citations were reviewed. To determine the origins of each recommendation further, we reviewed three documents predating the current guidelines: WHO’s 1981 severe protein-energy malnutrition recommendations,^9^ and two textbooks commonly used before the publication of WHO’s first severe malnutrition guidelines in 1999.^17^^,^^18^

According to formal GRADE (grading of recommendations, assessment, development and evaluation) assessment, each of the recommendations we investigated was of low, very low or unclassifiable quality. We evaluated each recommendation using GRADE’s directness assessment, as this provided meaningfully differentiated categories of evidence quality.^16^ Recommendations that were not supported by any cited evidence were considered to be based entirely on expert opinion. Recommendations were defined as indirectly supported if all of the cited studies were either among populations other than children with complicated severe acute malnutrition – e.g. HIV care guidance derived from studies of HIV-infected children without concurrent malnutrition – or only based on a treatment that was similar, but not identical, to the WHO recommended treatment – e.g. commercial ready-to-use therapeutic foods recommended on the basis of trials of locally produced versions of such foods. If at least one study concerning the endorsed intervention in a population of children with complicated severe acute malnutrition was cited in support of a recommendation, then that recommendation was considered to be directly supported. Direct evidence was further categorized as an observational study or a randomized trial.

To determine the aims and extent of any recently completed, ongoing, or pending trials relevant to the management of complicated severe acute malnutrition, we searched the WHO International Clinical Trials Registry Platform, the United States National Institutes of Health’s clinicaltrials.gov database and the Controlled Trials metaRegister systematically, using the search terms “malnutrition” and “wasting”. We completed this search on 10 August 2015. We fully reviewed all records with relevant or non-specific titles and included interventional trials among children with complicated severe acute malnutrition. We excluded trials already cited in WHO guidelines and those stopped before subject enrolment. We investigated the publication status and results of relevant trials by searching PubMed for the corresponding registration numbers.

## Results

**Fig. 1:**
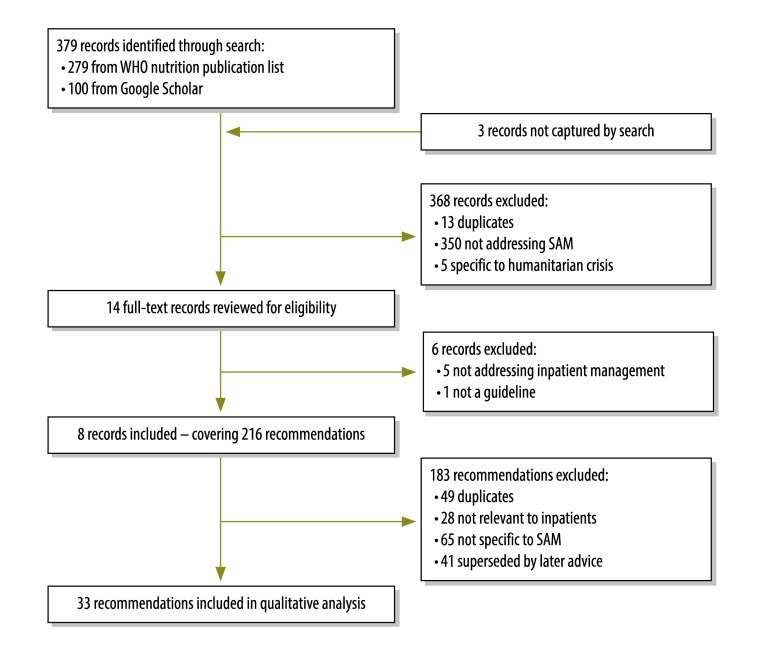
Flowchart of the search for guidelines and recommendations on the inpatient management of severe acute malnutrition, 2015

Eight documents containing 33 current recommendations met our inclusion criteria (Fig. 1).^2^^,^^9^^,^^10^^,^^17^^–^^20^ The lineage of the 33 recommendations is summarized in Table 1. Expert opinion, in the absence of published evidence, was the basis for 16 (48.5%) of the recommendations. Three (9.1%) and six (18.2%) of the recommendations were drawn from direct observational or indirect evidence, respectively. The remaining eight recommendations (24.2%) were each supported by at least one direct randomized trial.

SAM: severe acute malnutrition.

**Table 1:** Ancestry of evidence cited in support of the World Health Organization’s recommendations on the inpatient management of children with severe acute malnutrition

Recommendation	History			Evidence base, year published		
	First released	Last modified		Direct RCT	Direct observational	Indirect
**Micronutrients**						
200 000 IU of vitamin A for patients with eye signs of deficiency^a^	1981	–		1998, 2007, 2012	–	–
200 000 IU of vitamin A for patients with measles^1^	2003	–		1998, 2007, 2012	–	–
200 000 IU of vitamin A for patients not receiving vitamin A via feeds or other supplements^a^	2013	–		1998, 2007, 2012	–	–
5000 IU of vitamin A per day^a^	2013	–		1998, 2007, 2012	–	–
Zinc for patients with diarrhoea unless receiving zinc-fortified feeds	2013	–		–	–	–
No difference in zinc and vitamin A dosing based on HIV status^b^	2013	–		–	–	2010
Copper, folic acid, iron, magnesium and potassium to be given daily for at least 2 weeks	1992	1996		–	–	–
**Feeding**						
Feed immediately on admission, then every 2–3 hours. Transition from F-75 therapeutic milk feed to RUTF when patient stable, with appetite and decreasing oedema^c^	2003	–		–	1998	1989, 1998,^a^ 1998,^b^ 1998,^c^ 2009
Transition from F-100 therapeutic milk feed to RUTF when weight gain is rapid and patient accepting diet^c^	2003	–		–	1998	1989, 1998,^a^ 1998,^b^ 1998,^c^ 2009
For patient aged < 6 months, support breastfeeding – or relactate – with supplementary feeds and do not give undiluted F-100^d^	1981	2013		2009	2000	2009
No difference in feeding approach based on HIV status	2013	–		–	–	–
Can give RUTF in acute or persistent diarrhoea cases	2013	–		–	–	1994, 1995,1997, 2002, 2005
**Fluid management**						
Give ReSoMal for mild–moderate dehydration in non-cholera cases	1999	–		2003	2000	1999, 2000, 2001
Give standard low-osmolarity ORS for mild–moderate dehydration in suspected cases of cholera	2013	–		2009	–	–
For shock or severe dehydration, give intravenous Ringer’s lactate solution or half-strength Darrow’s solution, each supplemented with 5% dextrose^e^	1999	2013		2010	–	–
Every 5–10 minutes, monitor patients receiving intravenous fluids to check for overload	1999	–		–	–	–
Give blood transfusion, at 10 ml/kg, for shock if no improvement after 1 hour of intravenous therapy, and for severe anaemia	1999	–		–	–	–
Do not give blood transfusions > 24 hours post-admission	2013	–		–	2006	–
**ART**						
Start lifelong ART if patient aged < 24 months^9^	2013	–		–	–	2009, 2010
Start lifelong ART, based on CD4 counts or clinical staging, if patient aged ≥ 24 months^f^	2013	–		–	–	2009, 2010
Start ART after stabilization of complications	2013	–		–	–	2009, 2011, 2012
**Hypoglycaemia and hypothermia**						
If patient conscious, give 50 ml bolus of 10% dextrose – by mouth or nasogastric tube – then F-75 every 30 minutes for 2 hours	1969 or before	1996		–	–	–
If patient unconscious, lethargic or convulsing, give 10% dextrose intravenously, at 5 ml/kg, and then 50 ml of 10% dextrose by mouth	1969 or before	1996		–	–	–
**Infection**						
Give empiric ampicillin and gentamycin and then, if no response, chloramphenicol	1969 or before	1996		–	–	–
Patients aged < 6 months should receive same antibiotics as older children	2013	–		–	–	–
Give measles vaccine to non-immunized children aged ≥ 6 months	1996	–		–	–	–
**Discharge from inpatient or outpatient care**						
Transfer to outpatient care on clinical condition rather than anthropometry	2013	–		–	–	–
Move patients aged < 6 months to outpatient care if their daily weight gain exceeds the median growth velocity standard or is > 5 mg/kg/day for 3 days	2013	–		–	–	–
Discharge from outpatient care when WHZ is ≥ –2 or MUAC is ≥ 125 mm	2013	–		–	–	–
The anthropometric measure that qualified a child for admission should be used to monitor the child’s outpatient progress^g^	2013	–		–	–	–
If oedema was the only observed complication, normal anthropometrics can be used to monitor outpatient progress	2013	–		–	–	–
Discharge from outpatient care should not be based on percentage weight gain	2013	–		–	–	2004, 2012
**Emotional support**						
Provide patient with emotional and sensory support	1969 or before	–		–	–	–

ART: antiretroviral therapy; HIV: human immunodeficiency virus; IU: international unit; MUAC: mid-upper arm circumference; ORS: oral rehydration solution; RCT: randomized controlled trial; RUTF: ready-to-use therapeutic foods; WHZ: weight-for-height *z*-score.

^a^ All vitamin A recommendations are supported by the same randomized trials.

^b^ Citation for vitamin A and zinc dosing in HIV infection is a Cochrane review of five vitamin A and two zinc randomized trials indirectly related to the management of complicated severe acute malnutrition.

^c^ The F-75 and F-100 therapeutic milk feeding recommendations are supported by the same studies.

^d^ If maternal breastfeeding is not possible, wet nursing should be encouraged.

^e^ If neither solution available, use 0.45% saline with 5% dextrose.

^f^ Based on indirect evidence discussed in two sets of World Health Organization guidelines.^21^^,^^22^

^g^ That is, if the diagnosis was made on low MUAC, use MUAC – and not WHZ – to quantify recovery.

Twenty-three (69.7%) recommendations had been added or revised since the original guideline published in 1999.^10^ Only six (26.1%) of these 23 were supported by a directly relevant randomized trial. Three (13.0%) and six (26.1%) were supported by at least one direct observational or indirect study, respectively, while no references were cited in support of the remaining eight (34.8%) recommendations. The 1999 guidelines^10^ presented a 10-step management protocol – as originally proposed in the article *Ten steps to recovery* that was published in 1996.^11^ Five (15.2%) of the 33 current recommendations are identical to – or slight modifications of – the recommendations first proposed in this 1996 article. Seven (21.2%) of the current recommendations originated before 1996 – although five of these have since been slightly revised.

### Recommendation age and quality

The age of the recommendation and quality of supporting evidence varied according to the involved clinical area.

#### Micronutrients

Micronutrient recommendations were largely based on expert opinion, although three randomized trials^23^^–^^25^ directly supported two of the recommendations made in the 2013 update: low-dose vitamin A administration, reserving high-dose vitamin A for those with eye signs of deficiency or measles.^4^ Collectively, the trials demonstrated that either dose of vitamin A was superior to placebo, and that high-dose vitamin A offered no benefit compared with low-dose and might be associated with nosocomial diarrhoea and pneumonia. The 2013 update also recommended that HIV-infected children receive the same zinc and vitamin A doses as uninfected peers.^4^ This recommendation was supported by a systematic review of studies among HIV-infected children and adults without malnutrition, which indicated that HIV infection should not alter zinc requirements.^26^ Specific recommendations on the broader micronutrient package, which have remained constant for over 20 years, are all based on expert opinion.

#### Feeding

Indirectly related studies were the predominant reference type cited in support of the feeding recommendations. Recommendations for the use of therapeutic milk feeds – i.e. F-75 and F-100 – and the criteria for transition to ready-to-use therapeutic foods were last updated in 2003^2^ and were based on the results of six studies. Five of these studies demonstrated an association between refeeding syndrome and death among adolescents with eating disorders, children with neurological dysphagia, children with parent-imposed starvation, and critically ill adults in high-income settings.^27^^–^^31^ The 2013 update^4^ advised against use of undiluted F-100 among young infants, based on a direct study that indicated a possible connection between undiluted F-100 and renal solute overload, hypernatraemia and death.^32^ Specific advice on breastfeeding has remained largely unchanged for almost half a century.^18^

#### Fluid management

Three of the six recommendations on fluid management – including the specification of low-osmolarity salts for cholera – had been revised in the 2013 update.^4^ Recommendations for the treatment of shock or severe dehydration underwent a relatively minor re-ordering in the preference of intravenous fluids, based on a direct randomized trial of 62 children, in which Ringer’s lactate solution with 5% dextrose was compared with half-strength Darrow’s solution with 5% dextrose. Neither of these fluids was found to correct shock sufficiently and the choice of fluid had no significant effect on mortality.^33^ Finally, the study that was cited in support of limiting the timing, indications and infusion rates for transfusions demonstrated a strong association between mortality and transfusion – although adjustment for confounding by indication may have been insufficient.^34^

#### Antiretroviral treatment

Although three recommendations on antiretroviral treatment were added in the 2013 update,^4^ none was supported by direct evidence. Antiretroviral initiation recommendations referenced WHO’s guidelines on the management of childhood HIV infection.^21^^,^^22^ The advice to initiate antiretrovirals after clinical stabilization cited two pharmacokinetic studies among children with varying degrees of malnutrition^35^^,^^36^ and one retrospective study that demonstrated faster recovery when antiretroviral treatment was initiated within 21 days of the diagnosis of uncomplicated severe malnutrition.^37^

#### Other clinical problems

Recommendations on the management of hypoglycaemia, hypothermia and acute infections – including specifics related to antimicrobial treatment – were made in the *Ten steps to recovery* article.^11^ They remain unchanged and are not supported by any cited evidence.

#### Discharge and follow-up

Six recommendations on discharge from hospital and outpatient care were added in the 2013 update^4^ and were almost exclusively drawn from expert opinion. Supporting citations were limited to two indirect retrospective studies demonstrating that mid-upper arm circumference was an adequate measure of outpatient progress.^38^^,^^39^ The results of these studies led to the recommendation to eliminate percentage weight gain as a criterion for discharge from outpatient follow-up.

### Ongoing or recent trials

**Fig. 2:**
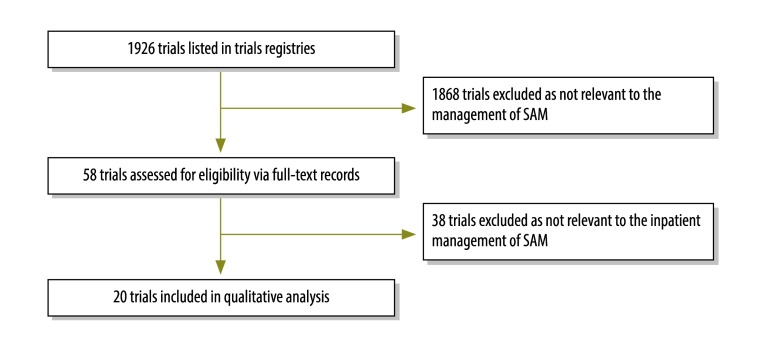
Flowchart of the search for recent or current trials relevant to the inpatient management of severe acute malnutrition, 2015

Our search of trials registries yielded the full records of 58 trials – after review of trial titles (Fig. 2). Twenty of these trials met our inclusion criteria (Table 2). Fifteen of the 20 trials had been completed – and the results of four had been published – by the time of our search.^40^^–^^43^ Two had reported statistically significant results; one demonstrated that community follow-up increased linear growth and clinic attendance^43^ and the other that long-chain *n*-3 polyunsaturated fatty acid in erythrocytes increased among severely malnourished children who were given ready-to-use therapeutic food enriched with polyunsaturated fatty acid.^42^ The other two published trials, which detected no significant differences, compared alternative formulations of ready-to-use therapeutic food with standard formulations. Of the 16 unpublished trials, nine and two had been designed to investigate alternative feeding regimens and the use of probiotics, respectively. One each had been designed to investigate pancreatic enzyme replacement, antioxidants, intravenous rehydration, stool output assessment, and antiretroviral pharmacokinetics. Three unpublished antibiotic trials – completed between 2008 and 2014 – examined ciprofloxacin pharmacokinetics, ceftriaxone for concurrent pneumonia, and post-discharge prophylaxis with co-trimoxazole (Table 2).

SAM: severe acute malnutrition.

**Table 2:** Registered clinical trials addressing the inpatient or post-discharge management of children with complicated severe acute malnutrition, 2015

Topic, title, country	Registry identifier	Date of last update^a^	Status^a^
**Antibiotics**			
Antibiotics in concurrent pneumonia, Bangladesh	NCT00968370	14 July 2013	Complete
Post-discharge co-trimoxazole prophylaxis, Kenya	NCT00934492	15 August 2014	Complete
Oral ciprofloxacin, Kenya	ISRCTN31079753	2 February 2009	Complete
**Antiretrovirals**			
Steady-state pharmacokinetics in concurrent HIV infection, Uganda, United Republic of Tanzania and Zimbabwe	NCT01818258	5 August 2015	Not yet recruiting
**Feeding**			
Comparison of RUTF with 10% and 25% milk, Malawi	ISRCTN54186063	4 June 2009	Complete^b^
Reformulated F-75 therapeutic milk feed, Kenya and Malawi	NCT02246296	6 January 2015	Ongoing
Rehabilitation with undiluted F-100 or diluted F-100, Bangladesh	NCT01558440	26 July 2015	Complete
RUTF based on sorghum, soybean and maize, Malawi	PACTR201505001101224	15 April 2015	Not yet recruiting
RUTF based on soybean, Bangladesh	NCT01634009	4 March 2015	Ongoing
RUTF enriched with n-3 PUFA, Kenya	NCT01593969	15 August 2014	Complete^b^
Three dietary regimes, Malawi	ISRCTN13916953	14 January 2013	Complete
Three new formulations of RUTF, Malawi	ISRCTN19364765	23 July 2009	Complete^b^
Whole milk during initial management, India	CTRI/2011/07/001853	3 May 2012	Complete
**Fluids**			
Slow versus rapid rehydration, Bangladesh	NCT02216708	20 August 2014	Complete
**Follow-up**			
Community-based follow-up, Bangladesh	NCT01157741	7 July 2010	Complete^b^
**Stool output**			
Stool frequency, Malawi	ISRCTN11571116	15 January 2014	Complete
**Supplements**			
Antioxidants and oxidants, Jamaica	NCT00069134	27 January 2015	Ongoing
Pancreatic exocrine replacement therapy, Malawi	ISRCTN57423639	14 April 2014	Complete
Probiotics in recovery, Uganda	ISRCTN16454889	12 May 2014	Complete
Spirulina supplementation, Niger	PACTR201406000810205	9 April 2014	Complete

HIV: human immunodeficiency virus; PUFA: polyunsaturated fatty acids; RUTF: ready-to-use therapeutic food.

^a^ As recorded on 10 August 2015.

^b^ Results published before 10 August 2015.

## Discussion

The 2013 update stated that “major research gaps were identified in each of the sections covered”.^4^ Our analysis shows that such gaps persist and extend across the entire spectrum of guidance on the management of complicated severe acute malnutrition. The absence of relevant published data has forced a reliance on expert opinion. The evidence that was cited in support of many recommendations was of very low quality and often did not specifically pertain to the recommended treatment. These deficits demonstrate that guideline reforms have been driven by an overwhelming clinical need – rather than by a body of compelling evidence. This is not criticism of WHO or the guidelines’ authors, who should be commended for creating pragmatic management documents by threading together the little solid evidence available and expert opinion.

It should be noted that recommendations supported by weak evidence or expert opinion are not necessarily incorrect. Many of the recommendations are grounded in the results of basic science research and careful clinical observations, much of which was made before the 1996 seminal *Ten steps to recovery* article. However, the population of paediatric inpatients with severe malnutrition has dramatically changed in the last 10–20 years. Over that period, HIV has emerged as an important contributing problem, younger infants have come to represent an increasing proportion of malnourished children and, for cases without complications, outpatient care has eclipsed hospital management.^3^^,^^4^ Data from previous eras may therefore not be generalizable to the modern child with complicated severe acute malnutrition.

In some areas the absence of clinical data is particularly concerning. For example, given that the largest burden of mortality from malnutrition is in sub-Saharan Africa, where the prevalence of HIV infection is relatively high, the lack of evidence to guide the management of HIV-infected children with malnutrition is worrying.^1^ A 2009 meta-analysis found that, among severely malnourished children, HIV infection was associated with a threefold increased risk of mortality.^6^ In a cohort study of severely malnourished children admitted to Queen Elizabeth Hospital in Malawi, HIV-infected children represented 64% of the deaths. The same study found that 67% of infants died.^3^ In the absence of data addressing these two populations – i.e. young infants and HIV-infected children with complicated severe malnutrition – the guidelines’ authors have been forced to generalize the management practices from other populations, without evidence that this is optimal or even appropriate.^4^ Furthermore, in the Malawian study, 25% of the children who were discharged died in the following 12 months and these deaths represented 44% of the total recorded mortality.^3^ Post-discharge mortality rates are high and their causes are poorly understood. This knowledge gap warrants urgent attention.

Antimicrobial therapy for severely malnourished inpatients represents another conspicuous knowledge gap. Empiric antibiotics have been recommended since at least 1969^18^ and the currently endorsed regimen has remained unchanged since it was standardized to ampicillin and gentamicin in 1996.^11^ A 1996 trial demonstrated the superiority of ampicillin and gentamicin compared with previously endorsed protocols relying on co-trimoxazole or penicillin and gentamicin.^44^We are not aware of any subsequent studies comparing the currently recommended regimen with other antimicrobials. In the care of severe acute malnutrition, fluid management also remains unresolved and understudied.

For ethical or practical reasons, some guidance areas are simply not amenable to clinical trials. Beyond vitamin A and zinc, the composition of the recommended micronutrient package has remained constant for decades and is underpinned by estimated daily requirements and observed micronutrient deficiencies in severe malnutrition. It would be impractical to run factorial trials for all micronutrients. However, this does not preclude the improvement of micronutrient packages via observational studies and targeted clinical trials, particularly given the etiological, environmental and social changes that have occurred in the decades since the foundational micronutrient research was conducted.

Our quantitative gap analysis builds on other reviews,^45^^,^^46^ including a 2012 systematic review^46^ – conducted in preparation for WHO’s 2013 update – that concluded: “For many of the most highly ranked questions evidence was lacking or inconclusive”.^4^ Our review of trials registries revealed that most of the upcoming, ongoing or recently completed relevant trials were focused on feeding and nutrition – areas not identified as priorities in the 2013 update^4^ or supporting systematic review.^46^ Furthermore, the areas in highest need of evidence – e.g. intravenous fluids, antimicrobials, the treatment of infants younger than six months and HIV-infected children, and post-discharge management – were very modestly represented. Therefore, it is unlikely that currently registered trials will address many of the critical knowledge gaps related to the inpatient management of severe acute malnutrition.

The paucity of relevant research may arise from a misconception that further work is unnecessary because adequate guidelines exist. As we have described, however, the underlying evidence for most management areas is weak. The current global undernutrition research agenda is largely focused on reducing the burden of stunting and moderate acute malnutrition – important and justifiable areas of concern. However, we should not neglect the half a million children who die from severe acute malnutrition annually.^1^

Epidemiological studies that define the etiologies of illness and mortality form an important foundation for interventional trials. Recent such studies have challenged long-standing beliefs about the relative importance of specific pathogens in pneumonia and diarrhoeal disease. For example, a South African study found cytomegalovirus, *Mycobacterium tuberculosis* and *Pneumocystis jiroveci* to be frequent causes of treatment failure among children with severe pneumonia^47^ while the Global Enteric Multicentre Study demonstrated *Cryptosporidium*, enterotoxigenic *Escherichia coli*, rotavirus and *Shigella* to be leading causes of childhood diarrhoeal death.^48^ These findings have spurred interventional trials that hopefully will improve management and save lives. A few studies have evaluated the causes of infection or death among children admitted for severe acute malnutrition.^49^^,^^50^ However, we are aware of no recent or robustly sampled investigation of the causes – including non-infectious etiologies – of mortality among such children during hospitalization or post-discharge. The failure of many trials to find statistically significant results may stem from a superficial understanding of the contemporary etiologies of such mortality. For example, if children with environmental enteric dysfunction require specific treatment more than children who are affected by food insecurity alone, then including both groups of children in trials of ready-to-use therapeutic foods could lead to attenuated estimates of efficacy and reduced statistical power. An improved understanding of the epidemiology of complicated severe acute malnutrition will facilitate the efficient design of clinical trials and catalyse the discovery of new and effective interventions.

This paper is not a detailed systematic review but rather a tracing of the lineage of each recommendation and its supporting citations. We did not review evidence that was not referenced in the relevant WHO guidelines. Early guidelines were published at a time when evidence citation was uncommon. Any relevant evidence available to these guidelines’ authors will not have been captured by our review unless cited in subsequent updates. Additionally, it is impossible to quantify the cumulative clinical experience of the many experts who have contributed to the guidelines. This paper does not address why severely malnourished children admitted to Asian hospitals seem to experience different mortality rates to their counterparts in African facilities. It would be useful to determine, in various settings, the proportion of cases of severe malnutrition that present with complications.

In conclusion, we found that the evidence base for the management of complicated severe acute malnutrition is heavily reliant on expert opinion in the absence of published data, that the relevant recommendations have undergone very limited substantive revision over the past two or more decades and that few ongoing clinical trials are being conducted in high priority areas. Although enhanced implementation of current guidelines would improve outcomes, a renewed and even modest investment in relevant epidemiological and clinical research is likely to lead to more effective recommendations and lower mortality.
